# Integration of Microfractionation, qNMR and Zebrafish Screening for the *In Vivo* Bioassay-Guided Isolation and Quantitative Bioactivity Analysis of Natural Products

**DOI:** 10.1371/journal.pone.0064006

**Published:** 2013-05-21

**Authors:** Nadine Bohni, María Lorena Cordero-Maldonado, Jan Maes, Dany Siverio-Mota, Laurence Marcourt, Sebastian Munck, Appolinary R. Kamuhabwa, Mainen J. Moshi, Camila V. Esguerra, Peter A. M. de Witte, Alexander D. Crawford, Jean-Luc Wolfender

**Affiliations:** 1 School of Pharmaceutical Sciences, EPGL, University of Geneva, University of Lausanne, Geneva, Switzerland; 2 Laboratory for Molecular Biodiscovery, Department of Pharmaceutical and Pharmacological Sciences, University of Leuven, Leuven, Belgium; 3 Faculty of Chemistry Sciences, School of Biochemistry and Pharmacy, University of Cuenca, Cuenca, Ecuador; 4 VIB Center for the Biology of Disease, University of Leuven, Leuven, Belgium; 5 Faculty of Pharmacy, Muhimbili University of Health and Allied Sciences, Dar es Salaam, Tanzania; University of New South Wales, Australia

## Abstract

Natural products (NPs) are an attractive source of chemical diversity for small-molecule drug discovery. Several challenges nevertheless persist with respect to NP discovery, including the time and effort required for bioassay-guided isolation of bioactive NPs, and the limited biomedical relevance to date of *in vitro* bioassays used in this context. With regard to bioassays, zebrafish have recently emerged as an effective model system for chemical biology, allowing *in vivo* high-content screens that are compatible with microgram amounts of compound. For the deconvolution of the complex extracts into their individual constituents, recent progress has been achieved on several fronts as analytical techniques now enable the rapid microfractionation of extracts, and microflow NMR methods have developed to the point of allowing the identification of microgram amounts of NPs. Here we combine advanced analytical methods with high-content screening in zebrafish to create an integrated platform for microgram-scale, *in vivo* NP discovery. We use this platform for the bioassay-guided fractionation of an East African medicinal plant, *Rhynchosia viscosa*, resulting in the identification of both known and novel isoflavone derivatives with anti-angiogenic and anti-inflammatory activity. Quantitative microflow NMR is used both to determine the structure of bioactive compounds and to quantify them for direct dose-response experiments at the microgram scale. The key advantages of this approach are (1) the microgram scale at which both biological and analytical experiments can be performed, (2) the speed and the rationality of the bioassay-guided fractionation – generic for NP extracts of diverse origin – that requires only limited sample-specific optimization and (3) the use of microflow NMR for quantification, enabling the identification and dose-response experiments with only tens of micrograms of each compound. This study demonstrates that a complete *in vivo* bioassay-guided fractionation can be performed with only 20 mg of NP extract within a few days.

## Introduction

Natural products (NPs) are an important source of drug-like compounds for the discovery of new therapeutic candidates and over time their chemical diversity has contributed significantly to the development of drugs for a wide range of diseases. The majority of new drugs approved within the last thirty years are either natural products themselves or are derived from natural products [1]–[3].

Currently, most drug discovery programs are based on high-throughput screening (HTS) to rapidly query the bioactivity of large libraries of synthetic compounds. In contrast, the isolation and characterization of bioactive secondary metabolites present in complex NP extracts involves the application of several complementary methodologies that require considerably more time and effort [4], [5]. In addition, there are several inherent caveats associated with testing NPs in HTS. Crude extracts from various species of plants, fungi, and bacteria, herein after called NP extracts, are complex mixtures of mostly uncharacterized compounds, some of which might have undesired effects. The chemical properties of certain secondary metabolites might hinder the test readout and interfering constituents present in the crude extract can either mask the biological activity [6] or cause toxic effects that lead to false positives, e.g. in enzymatic assays. Nevertheless, a considerable advantage of NPs is their chemical diversity. The chemical space occupied by NPs is different from the one occupied by synthetic compounds – often with far greater degrees of 3-dimensionality and structural complexity. NPs are a promising source of diverse molecular scaffolds for the discovery of novel lead compounds against original targets [7] and recently, combinatorial libraries with NP-like compounds have been used for HTS [3].

Bioassay-guided fractionation has proven successful as a well-established platform to isolate and characterize active constituents present in NP extracts, which are then suitable for HTS [8], [9]. However, such an approach requires multiple chromatographic steps and large amounts of biological material. Recent technological improvements in the area of chromatographic separation methods have nevertheless provided new possibilities to accelerate the overall process of bioassay-guided fractionation. In particular, the development of microfractionation approaches based on advanced high performance liquid chromatography (HPLC) techniques is now enabling the systematic separation of complex plant extracts using more widely applicable protocols [10]. The increasing sophistication of such techniques by linking them directly (on-line) or indirectly by adding an additional step of sample concentration (at-line) with analytical assays allows the more rapid dereplication of extracts – identifying known NPs prior to thorough characterization – thereby focusing resources on novel molecules.

Although active constituents present in NP extracts can now be identified more quickly as less time is expended on the purification of inactive constituents, still appreciable amount of time is invested if the bioactive compounds need to be isolated for the determination of their structure and in-depth biological testing. This is the bottleneck of bioassay-guided isolation since the *de novo* structure elucidation of small molecules relies on NMR spectroscopy, which has intrinsically low sensitivity. Nevertheless, with the emergence of microflow NMR [11] and cryo and microcryo NMR technologies [12]–[14] used routinely in NP drug discovery, the boundaries could be pushed to the low microgram scale of sample needed for the acquisition of ^1^H-^13^C and ^13^C spectra.

When working with HPLC-based biological profiling, another issue is to quantify the potency of a given extract constituent in a given bioassay since the microgram quantities obtained by microfractionation have to be correctly estimated [15]. Weighing of the individual microfractions is not only impractical but also inaccurate at sub-milligram quantities. Furthermore, compound purity is not taken into account. Since NMR gives an absolute signal response, it can not only provide unambiguous compound identification but allows precise quantification even of unknown compounds and estimate ratios in fractions still containing mixtures. NMR quantification can be performed either with an internal standard, using the ERETIC (electronic reference to access *in vivo* concentrations) [16] method that demands specialized electronic equipment, or the PULCON (pulse length based concentration determination) [17] method with reference to an external standard.

The ultimate impact of these new methods on the field of NP discovery, however, will be determined by the bioassays with which they can be combined. The recent report of a microfractionation approach involving the coupling of microbore HPLC separation with an at-line 1536-well biochemical screening assay for protein kinase A activity assessment and with parallel QTOF MS (quadrupole time of flight MS) data acquisition for analyte identification is an excellent example of the potential of this technology [18].

Despite its utility for HTS of active compounds, the reliance of such strategies on enzymatic or *in vitro* cell-based assays to assess their biological activity limits the biomedical relevance of the active metabolites isolated in this manner. By combining high-resolution microfractionation with high-content assays, the activity of the separated constituents would be analyzed and validated to an appreciably higher degree.

In contrast with enzymatic or cell-based reporter assays, high-content bioassays (e.g. phenotypic assays using cells or organisms) allow the unbiased analysis of pharmacological activity. In particular, *in vivo* animal models offer the possibility to screen for biomedically relevant bioactivities in a target- and pathway-independent manner. Nevertheless, mammalian models such as rodents require larger amounts of compound (in the milligram range) for activity analysis, and are therefore not ideal *in vivo* platforms for rapid HPLC profiling and microfractionation strategies.

In this context, zebrafish bioassays represent an attractive alternative to determine the *in vivo* bioactivity of chromatographic fractions containing only microgram amounts of individual compounds. Zebrafish – *Danio rerio* – have recently emerged as a reliable *in vivo* vertebrate model system for functional genomics and drug discovery [19]. Beyond their many physiological and pharmacological similarities to mammals, zebrafish have important advantages such as high fecundity (up to hundreds of offspring per day), the small size of embryos and larvae (0.5 to 5 mm depending on the developmental stage), optical transparency and rapid development *ex utero*. These features confirm zebrafish as a versatile *in vivo* experimental model compatible with HTS and microfractionation techniques in the field of NP discovery [20]. In this regard, the amenability of using zebrafish embryos and larvae in microtiter plates (96- and even 384- well design) allows early *in vivo* analysis of the activity of small-molecule compounds isolated by microfractionation approaches. Depending on the potency of these isolated compounds, the requirement of only microgram amounts to induce an initial biological response represents another excellent benefit of using zebrafish as a model organism over other higher vertebrates (e.g. rodents, in which the active dose requirements are usually a thousand-fold higher [21]).

This latter feature is key for NP discovery, as many high-resolution separation methods based on HPLC, particularly microfractionation, result in very limited amounts of samples that would otherwise be insufficient for the *in vivo* analysis of activity.

In this study we combine HPLC profiling with microfractionation and sensitive microflow NMR at-line detection with a high-content *in vivo* screen in zebrafish for the rapid identification of bioactive NPs in crude plant extracts as well as for the direct estimation of their biological activity and potency at the microgram level. We illustrate this approach by investigating both the anti-inflammatory and the anti-angiogenic activity of a Fabaceae plant used in traditional Tanzanian medicine, *Rhynchosia viscosa* (Roth) DC. Optimization of the workflow with minimal amounts of extract was successfully achieved providing a generic approach that is adaptable for any other sample, even if extracts are only available in milligram amounts (e.g. because the phytochemical analysis is done on a herbarium sample, supply of the extract is difficult or the biological species under investigation is small in size).

## Results and Discussion

### Anti-inflammatory and Anti-angiogenic Activity of *Rhynchosia viscosa* in Zebrafish

**Figure 1:**
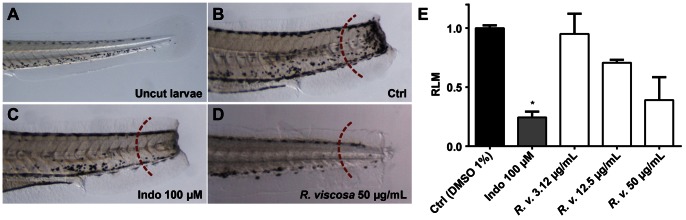
Anti-inflammatory activity of the methanolic extract of *Rhynchosia viscosa.* Anti-inflammatory activity was determined in an acute inflammation assay based on tail transection and treatment with lipopolysaccharides (LPS). **A** to **D**, zebrafish larvae are 4 days post-fertilization (dpf) with anterior to the left, scale bar = 10 µm. After tail transection and LPS exposure, stained leukocytes appear as black-brown spots migrating to the injured area in the transected tails. Migrating leukocytes were counted on one side in the tail in the region to the right of the dashed red arc and migration values were expressed as relative leukocyte migration (RLM) (**E**). **A**, tail of an uncut larva; **B**, negative control (DMSO 1%); **C**, positive control (indomethacin 100 µM) **D**, crude extract of *R. viscosa* at 50 µg/mL; **E**, graph displaying the RLM of 4 dpf larvae (n = 10) subjected to tail transection and incubation with *R. viscosa*. RLM ≤0.5 was established as cutoff for anti-inflammatory activity. * *p*<0.05.

Using a zebrafish-based inflammation assay [22], we screened crude methanolic extracts from over 80 East African medicinal plants. The extract of *Rhynchosia viscosa* (Roth) DC. (Fabaceae) inhibited leukocyte migration in tail-transected four days post-fertilization (4 dpf) larvae in a concentration-dependent manner (Figure 1). The anti-inflammatory effect of the crude extract of *R. viscosa* was evident at 50 µg/mL – a concentration at which a relative leukocyte migration (RLM) value of 0.39 was achieved (Figure 1D), in comparison with an RLM of 0.24 achieved by 100 µM indomethacin as a positive control (Figure 1E). Interestingly, the ethnomedicinal use of *R. viscosa* in Tanzania (local name: mfundofundo) includes the topical treatment of inflammatory skin disorders and insect bites (M. J. Moshi, personal communication), prompting us to perform follow-up studies for the identification of anti-inflammatory constituents of this plant.

**Figure 2:**
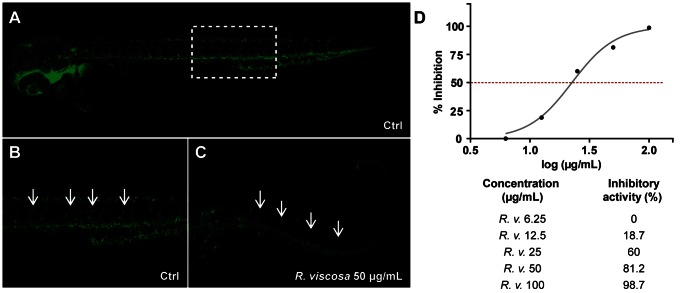
Anti-angiogenic activity of the methanolic extract of *Rhynchosia viscosa.* Inhibition of vascular outgrowth was determined in *fli-1*:EGFP transgenic embryos. At 16 hours post-fertilization (hpf), embryos were incubated with different concentrations of the methanolic extract of the plant and anti-angiogenic effects were assessed at 48 hpf. **A** to **C**, all embryos are 48 hpf, with anterior to the left, scale bar = 10 µm. **A**, untreated control (DMSO 1%); **B**, zoom of **A** (dashed box) showing normal outgrowth of intersegmental vessels (ISV) along the trunk of the larva (arrows); **C**, embryo treated with 50 µg/mL crude methanolic extract of *R. viscosa*. Inhibition or reduction of ISV growth is observed along the trunk (arrows); **D**, IC_50_ curve and values showing the inhibitory activity of the methanolic extract of *R. viscosa*.

In parallel, we also screened the extracts of these East African medicinal plants for their capacity to inhibit angiogenesis, based on their ability to restrict vascular outgrowth in *fli-1*:EGFP transgenic zebrafish embryos [23], which exhibit vasculature-specific expression of enhanced green fluorescent protein (EGFP) during embryonic and larval development. In addition to the identification of *Oxygonum sinuatum* (Meisn.) Dammer (Polygonaceae) and *Plectranthus barbatus* Andrews (Lamiaceae) as anti-angiogenic extracts [24], we found that the methanolic extract of the aerial parts of *R. viscosa* inhibited intersegmental vessel (ISV) outgrowth in *fli-1*:EGFP embryos in a concentration-dependent manner (Figure 2). In order to rapidly localize the compounds responsible for the bioactivity, high-resolution HPLC-based bioassay-guided fractionation of the extract was performed using the zebrafish vascular outgrowth assay given its higher throughput and lower assay volume compared to the lipopolysaccharide (LPS)-enhanced leukocyte migration assay.

### Generic Chromatographic Procedure for Optimal One-step Microfractionation of NP Extracts for the Rapid Localization of Bioactive Constituents

**Figure 3:**
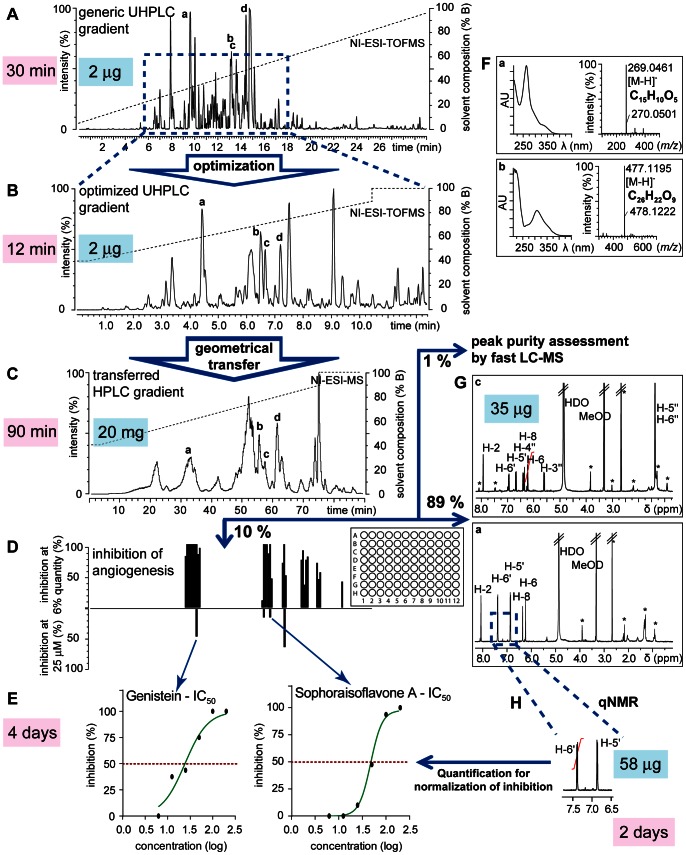
Generic procedure for the rapid identification of bioactive constituents from medium polar plant extracts. **A**, Generic ultra high pressure liquid chromatography – photo diode array – time of flight mass spectrometry (UHPLC-PDA-TOFMS) chromatogram. UHPLC conditions: Acquity BEH C_18_ column (150×2.1 mm i.d., 1.7 µm); A: 0.1 vol% formic acid (FA)-H_2_O, B: 0.1 vol% FA-acetonitrile, 5–95% B in 30′; 0.46 mL/min; ESI-MS detection in negative ion (NI) mode; **B**, Optimized UHPLC-PDA-TOFMS chromatogram for methanolic extract of *R. viscosa*. UHPLC conditions: Acquity BEH C_18_ column (100×2.1 i.d., 1.7 µm); A: 0.1 vol.% FA-H_2_O, B: 0.1 vol% FA-methanol (MeOH), 40–90% in 11.4′; 0.306 mL/min, ESI-MS detection in NI mode; **C**, Semi-preparative high performance liquid chromatography (HPLC) chromatogram for the microfractionation of the enriched extract of *R. viscosa*. HPLC conditions: XBridge™ BEH C_18_ column (250×10 mm i.d., 5 µm); A: 0.1 vol.% FA-H_2_O, B: 0.1 vol% FA-MeOH, 40–90% in 74.9′; 2.3 mL/min; ESI-MS detection in NI mode. The chromatographic gradient is geometrically transferred using mathematical models to obtain a comparable elution of extract constituents. Fractions were collected every 30 s directly into 96-deepwell plates. The so generated microfractions were aliquoted for anti-angiogenic screening (10% aliquot A), for fast LC-MS analysis (1%, aliquot B), and for NMR analysis (89%, aliquot C); **D**, Anti-angiogenic screen on 180 microfractions generated by microfractionation. Positive bars show inhibition of angiogenesis of microfractions tested at high concentration; negative bars show inhibition of angiogenesis of selected microfractions at 25 µM. The concentration was determined by quantitative NMR (qNMR) (**H**); **E**, Determination of IC_50_ using the quantitative information obtained by qNMR (**H**); **F**, On-line PDA and high-resolution MS information from (**A**) for the dereplication of plant constituents; **G**, ^1^H NMR spectra using the CapNMR™ probe for structure confirmation of bioactive constituents; **H**, Integration of well resolved aromatic protons for quantification of bioactive constituents to establish the potency of the anti-angiogenic and anti-inflammatory activity of the targeted compounds (**D**, **E**).

For the rapid isolation and identification of the bioactive constituents of *R. viscosa* we developed a generic chromatographic procedure which combines (1) ultra high pressure liquid chromatography – photo diode array – time of flight mass spectrometry (UHPLC-PDA-TOFMS) for extract profiling, (2) gradient transfer for one-step separation on semi-preparative HPLC and (3) microfractionation for a rapid collection of all LC peaks for further bioactivity assessment (Figure 3).

### UHPLC-PDA-TOFMS Profiling and Dereplication

Initially, a metabolite profiling at the analytical scale was performed with microgram amounts of crude extract on UHPLC-PDA-TOFMS to evaluate the extract complexity. This method combines high-resolution separation on sub-2 µm particle columns with high-resolution MS detection, which provides molecular formula information for all analytes on-line [25]. For this generic profiling, the separation was achieved on an enriched extract with optimal conditions for maximal peak capacity [26] (Figure 3A). The metabolite profiling revealed a large number of detected peaks to have PDA spectra corresponding to polyphenols with molecular weights ranging from 250 to 450 Da. Most of the PDA spectra were characteristic for either flavones or isoflavones, both known to be present in the Fabaceae family [27]. The high-resolution MS data gained from the UHPLC-PDA-TOFMS analysis provided molecular formula information for all detected LC peaks giving a first overview of the extract composition. This preliminary structural information was later used in combination with the bioassay results for the dereplication of the bioactive constituents.

### Determination of Generic Parameters for Microfractionation

In order to rapidly determine which compounds were responsible for the bioactivity of the enriched extract, a microfractionation strategy was developed to enable the acquisition of fractions in 96-well plate format with sufficient quantities both for bioactivity testing (anti-angiogenic assay) and for structural elucidation (high-resolution MS and ^1^H NMR analysis) of the bioactive compounds at the analytical level, starting with only a few milligrams of extract. According to the sensitivity of the zebrafish anti-angiogenic assay which was deduced from several known anti-angiogenic compounds with a range of *in vivo* potencies in zebrafish including SU5416 and emodin [24], it was estimated that the microfractionation procedure should yield at least 1 µg per well for an initial tracking of the anti-angiogenic activity over the entire chromatogram. On the other hand, since compound identification was foreseen based on microflow ^1^H NMR, it was necessary to keep a minimum of 5 µg for further dereplication. In addition, as the bioassay is carried out in a 96-well plate format that includes controls, the number of fractions had ideally to be 90 or a multiple thereof.

In order to obtain 5–10 µg per microfraction, it was estimated that 1.5 mg of enriched extract would be required. The loading was multiplied by a factor of 10 to ensure that most of the activity could be assessed and minor bioactive constituents could be detected, factoring in the recovery of a given metabolite through microfractionation on reversed phase (RP) columns is ∼ 70% (see Text S1). It was thus estimated that 20 mg of enriched extract would be sufficient for the entire microfractionation procedure and a column with an adapted loading capacity was selected. To minimize sample handling, fractions were collected directly into 96-deepwell plates, facilitating the subsequent drying of all samples at once by vacuum centrifugation, whereas a maximum volume of 1.2 mL of eluent per well had to be respected.

A column geometry of 250×10 mm was found to be a good compromise between loading capacity, HPLC resolution and microfraction volumes. In order to fill the deepwells with adequate eluent volumes and collect peaks with sufficient resolution, a fraction collection time of 30 sec and a flow rate of 2.3 mL/min were chosen.

Based on the gradient time constraints of the microfractionation procedure (90 min×180 microfractions), corresponding gradient time and flow rate were calculated for the analytical UHPLC (gradient time 11.4 min, flow rate 306 µL/min). This was necessary to optimize the gradient for the separation of the NPs in a specific extract at the analytical scale. For a good predictability of the separation efficiency between UHPLC and semi-preparative HPLC, the same phase chemistry and columns geometries with similar theoretical peak capacities [28] were chosen (see Materials & Methods).

All of these steps are generic, as the procedure is adaptable for any medium-polar extract compatible with RP separation.

### Separation Optimization Specific to *Rhynchosia viscosa* and Microfractionation

Since all generic parameters were fixed by the requirements of the bioassay and the structure identification, only the solvent system and the gradient needed to be adapted for profiling. Therefore, the chromatographic gradient method for the microfractionation was optimized on UHPLC-PDA-TOFMS by adapting the generic profiling gradient to maximize mixture component resolution over the run time allowed by the collection. In the case of *R. viscosa*, a linear gradient from 40% to 90% methanol (MeOH) was optimal (Figure 3B) (see Materials & Methods). This gradient was directly transferred to the semi-preparative system. The enriched extract (19.8 mg) was chromatographed in one step (Figure 3C) and 180 microfractions were generated and collected into 96-deepwell plates. Each microfraction (1.15 mL total volume) was divided into three aliquots: for the zebrafish angiogenesis assay (115 µL, 10% of the total volume, aliquot A); for LC-MS analysis (11.5 µL, 1% of the total volume, aliquot B); and for microflow NMR analysis (ca. 1.12 mL, 89% of the total volume, aliquot C).

### Anti-angiogenic Screen of Microfractions

Microfractions were screened for anti-angiogenic activity using the zebrafish-based vascular outgrowth assay described above. In an initial screen, 60% of each aliquot A (equivalent to 70 µl of the original 115 µl) was used. Inhibition was observed as the absence or reduction of vascular outgrowth. Microfractions inducing complete inhibition of vascular outgrowth or embryonic toxicity were tested at one third of this concentration (20% of each aliquot A, equivalent to 23 µl of the original 115 µl). This *in vivo* biological profiling revealed six main chromatographic zones containing anti-angiogenic compounds at high concentration (30.0–33.0 min, 54.5–55.0 min, 56.5–57.0 min, 60.5–61.0 min, 66.5–68.5 min, 71.5–72.5 min and 79.5 min) (Figure 3D). When testing at the lower concentration, only four zones (30.0–33.0 min, 54.5–55.0 min, 56.5–57.0 min and 60.5–61.0 min) were still active (data not shown). To rapidly identify the constituents responsible for the anti-angiogenic activity and to estimate the amount tested in the corresponding microfractions, ^1^H NMR spectra were recorded using microflow NMR.

### Rapid Compound Identification in Bioactive Microfractions

**Figure 4:**
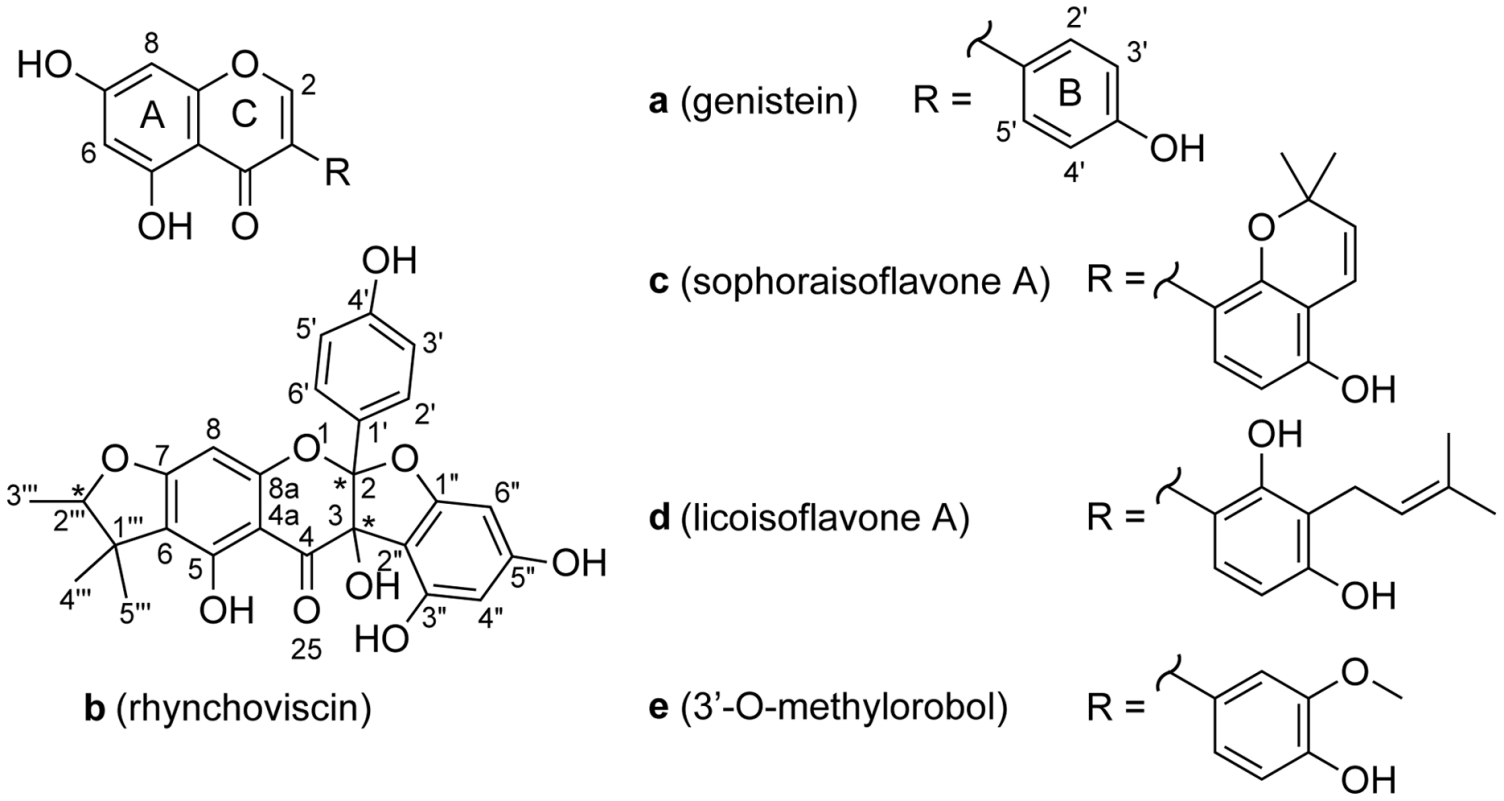
Anti-angiogenic constituents of methanolic extract of *Rhynchosia viscosa*. Compounds **a** and **c** exhibit anti-angiogenic and anti-inflammatory activity.

In the first active chromatographic zone, ten consecutive microfractions were found to inhibit angiogenesis (80–100% inhibition of vascular outgrowth at high concentration). The MS data recorded during microfractionation indicated a nominal mass of *m/z* 269 [M-H]^–^ for the main compound eluting in this region. The corresponding exact mass recorded during the UHPLC-PDA-TOFMS profiling of the extract was *m/z* 269.0461 (compound **a**) indicative of the molecular formula C_15_H_10_O_5_ (calc. *m/z* 269.0450, Δ 4.1 ppm). This was also validated by application of heuristic filtering [29], [30] (see Materials & Methods). A cross search with this molecular formula and with chemotaxonomic information (Fabaceae, Leguminosae) in the Dictionary of Natural Products (DNP) [31] revealed that **a** could correspond to 7,3′,4′-trihydroxyflavone or 5,7,4′-trihydroxyisoflavone (genistein). In addition, the PDA spectrum presented an absorption maximum (λ_max_) at 260, 290 sh and 325 sh nm characteristic for isoflavones such as genistein. Compound **a** was easily confirmed to be genistein (Figure 4) by the comparison of the ^1^H NMR spectrum of the corresponding microfraction obtained by microflow NMR (CapNMR™) with literature values [32]. In all the fractions collected in the 30.0–33.0 min region, the ^1^H signals of genistein were present confirming it to be responsible for the *in vivo* anti-angiogenic activity observed (Figure 3D).

The last two microfractions in this first zone contained another constituent with *m/z* 299.0549 (compound **e**) consistent with the molecular formula C_16_H_12_O_7_, and possibly another isoflavone derivative based on the dereplication by TOFMS and PDA (calc. *m/z* 299.0556, Δ 2.3 ppm, λ_max_ 260, 290 sh, 340 sh nm). The identification of this isoflavone was based on interpretation of the corresponding additional ^1^H signals to those of genistein in this microfraction. The presence of a methoxy substituent (δ 3.90) was revealed and its position at C-3′ was confirmed by comparison with reported data [33]. The molecule was finally identified as 3′-O-methylorobol. Further bioactivity analyses were not undertaken for this constituent as the molecule was not isolated as a pure compound but only in a mixture with genistein.

In the second active zone of the chromatogram, the two microfractions contained one single constituent (compound **b**) with *m/z* 477.1195 ([M-H]^–^ C_26_H_22_O_9_, calc. *m/z* 477.1186, Δ 1.9 ppm). A database search yielded six NPs with this molecular formula but none were isolated from Fabaceae species, nor were they consistent with the ^1^H NMR spectrum of **b**. The complete structure of this polyphenol could not be determined *de novo* only based on these data. The compound was named rhynchoviscin and its full structural identification is discussed below in the section “*De novo* identification of the novel compound **b**”.

In the third zone, the two microfractions contained another constituent (compound **c**) with *m/z* 351.0886 ([M-H]^–^_,_ C_20_H_16_O_6_, calc. *m/z* 351.0869, Δ 2.0 ppm) and with aromatic ^1^H signals typical of an isoflavone. This molecular formula matched with more than 100 possibilities in DNP and no hypothesis could be deduced. The ^1^H NMR spectrum in deuterated methanol (methanol-*d_4_*) was consistent with the configurational isomers licoisoflavone B and sophoraisoflavone A. An additional experiment by re-dissolution of the microfraction in acetone-*d_6_* confirmed that it was sophoraisoflavone A (Figure 4) by comparison of the ^1^H chemical shift of 5-O*H* (δ 13.07) [34].

In the fourth zone, two microfractions contained one major constituent (compound **d**) with *m/z* 353.1037 ([M-H]^–^, C_20_H_18_O_6,_ calc. *m/z* 353.1025, Δ 3.4 ppm) consistent with prenylated isoflavone derivatives. Beside the aromatic protons characteristic for isoflavones, ^1^H signals characteristic for a prenyl group were detected (two methyl signals (δ 1.66 and 1.77) correlating to a vinyl proton (δ 5.25) and further connected to a downfield-shifted methylene group (δ 3.38), as determined by 2D NMR). Comparison of chemical shifts with literature data [35] confirmed **d** to be licoisoflavone A (Figure 4).

At the high concentration, three more active zones were detected for compounds eluting after 66 min (Figure 3D). No exploitable NMR spectra could be recorded (no aromatic signals were detected) in the corresponding microfractions, and the activity was not seen when tested at the low concentration. These microfractions were not further studied.

### Quantification of Bioactive Molecules and Correlation with Anti-angiogenic Activity

To rapidly evaluate the potency of the bioactivity measured, a reliable estimation of the concentration present in each tested microfraction was made. In order to be generic and not have to depend on standards, NMR was used for quantification. Microflow NMR was found to be well-suited for the limited sample amounts present in the microfractions.

### Quantitative Microflow NMR

For NMR quantification a strategy that does not alter the sample by addition of an internal standard was favored so that any interference with bioassays is avoided. In this respect, a quantitative NMR (qNMR) method using an external calibration (PULCON [17]) was used. Further information on PULCON and the validation of the qNMR method are given in the Text S2.

Overall, the microflow qNMR method (1) provides a universal detection, (2) provides accurate estimation of sample amount in the microgram range without need of any reference compounds, and (3) is compatible with *in vivo* bioassays enabling fast and reliable identification of bioactive NPs.

### Quantification of Bioactive Constituents of *Rhynchosia viscosa*

The optimized qNMR parameters were used for the acquisition of the ^1^H NMR spectra of *R. viscosa* and thus, within the same experiment, both identification and quantitative information could be obtained for all microfractions displaying anti-angiogenic activities. The proton signal chosen for quantification of all the polyphenols corresponded to an aromatic proton signal on cycle B well isolated from interfering signals (Figure 3H). Quantifiable amounts were between 3 and 90 µg per microfraction. A maximum analysis time of 50 min (128 transients) was found to be a good compromise between throughput and detection limits.

For the bioactive compounds (**a** to **d**), the microfractions containing the greatest amounts were the following: **a** (32.5 min, 87 µg), **b** (54.5 min, 50 µg), **c** (56.5 min, 35 µg), **d** (61.0 min, 55 µg). These sub-milligram amounts could be readily converted into precise concentrations for determination of IC_50_ values in the bioassays, since molecular weight in each case was known from the LC-MS results. Thus, even at this stage, a good estimation of the bioactive potency of the unknown compound **b** could be established.

### Assessment of the Purity of Microfractions by Fast UHPLC-PDA-TOFMS

Prior to bioassay analysis and in parallel to NMR analysis, the purity of the microfractions selected for IC_50_ measurements was also determined using a fast UHPLC-PDA-TOFMS analysis using aliquot B kept from the microfractionation (see above). This revealed that the microfractionation generated always at least one microfraction containing only one constituent for compounds **a** to **d**. This also validates the reasoning to choose a collection strategy of 30 sec per microfraction.

This indicated that the strategy chosen was able to rapidly generate pure microfractions with well-defined quantities of compounds to be evaluated biologically in the low microgram range.

### Anti-angiogenic and Anti-inflammatory Activity of Compounds a to d

In the initial screen of the microfractions, a rapid localization of the bioactive constituents in the extract could be efficiently established (Figure 3D). This screen, however, provides information on how the initial activity of the extracts is distributed among its constituents based on their relative abundance in the extract. Now, since the purity and the amount of each compound in each microfraction is known from qNMR and MS analysis, a reliable evaluation of the potency of the activity could be performed for the determination of IC_50_ values.

**Figure 5:**
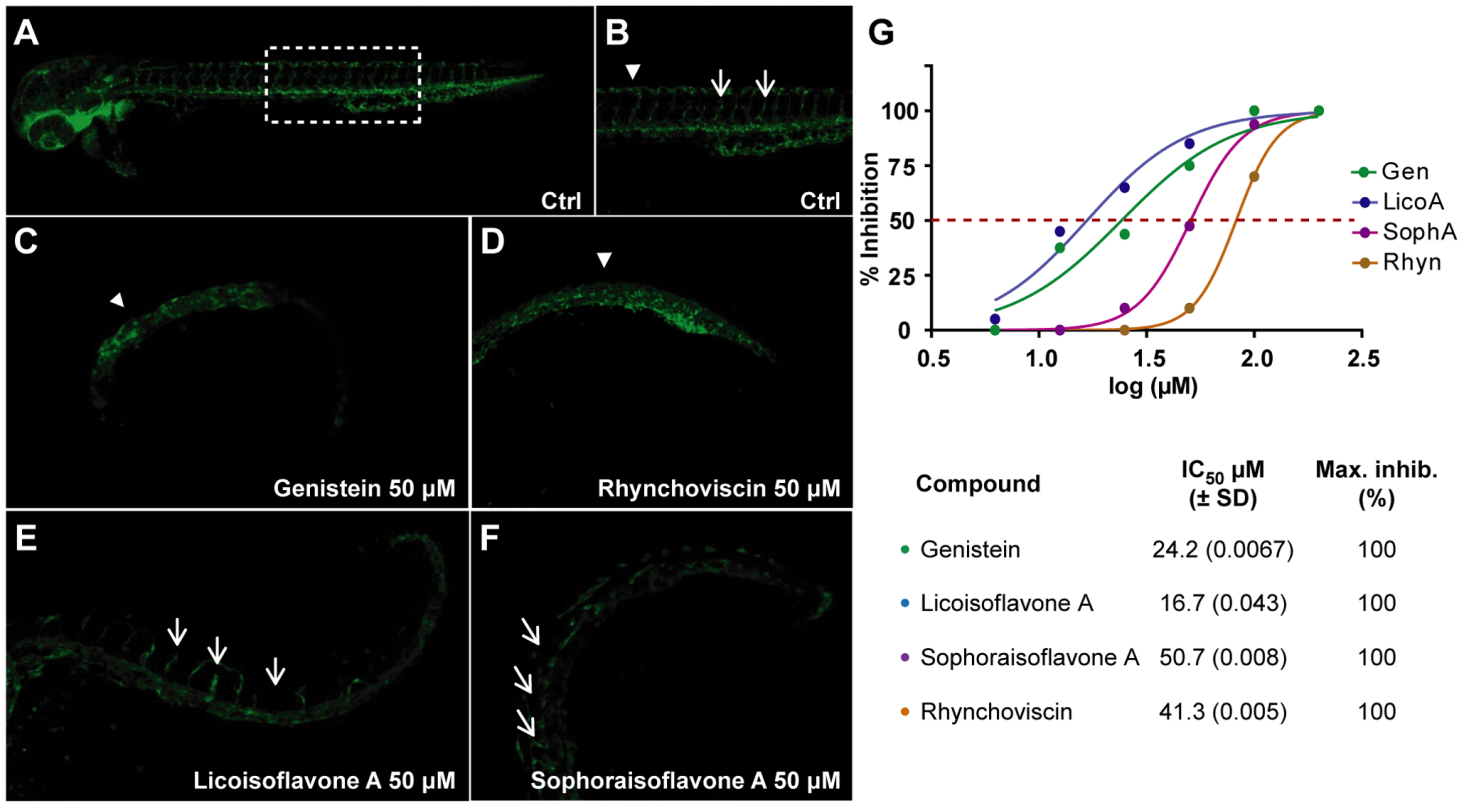
Bioactive compounds of *Rhynchosia viscosa* in the vascular outgrowth assay. IC_50_ curves and values were determined for each of the bioactive constituents of the methanolic extract of *R. viscosa*. Each compound, at six different concentrations, was assessed for their effect in the inhibition of intersegmental vessel (ISV) growth. **A** to **F**, all embryos are 48 hours post-fertilization (hpf), with anterior to the left, scale bar = 10 µm. **A**, untreated control (DMSO 1%); **B**, zoom of **A** (dashed box) showing normal outgrowth of intersegmental vessels (ISV) along the trunk of the larva (arrows); **C**, embryo treated with 50 µM genistein; **D**, embryo treated with 100 µM rhynchoviscin; **E**, embryo treated with 50 µM licoisoflavone A; **F**, embryo treated with 50 µM sophoraisoflavone. Arrowheads point the interconnection zone between the dorsal aorta and the posterior cardinal vein, arrows point the ISV; **G**, IC_50_ curves and values (µM) for each of the bioactive compounds of *R. viscosa*.

For this, aliquot C of each microfraction (89% of the original 1.15 ml, which was previously used for NMR analysis) was recovered and used to make a fixed-concentration solution in dimethyl sulfoxide (DMSO) to perform a concentration-response analysis and determine IC_50_ values for anti-angiogenic activity for compounds **a** to **d**. Genistein (**a**) and licoisoflavone A (**c**) displayed similar levels of potency, with IC_50_ values of 24.2 µM and 16.7 µM, respectively. Sophoraisoflavone A (**d**) and rhynchoviscin (**b**) were less potent but still clearly anti-angiogenic, with IC_50_ values of 50.7 µM and 41.3 µM, respectively (Figure 5). All four compounds phenocopied the anti-angiogenic effects of the *R.* *viscosa* extract in this assay (Figure 2).

**Figure 6:**
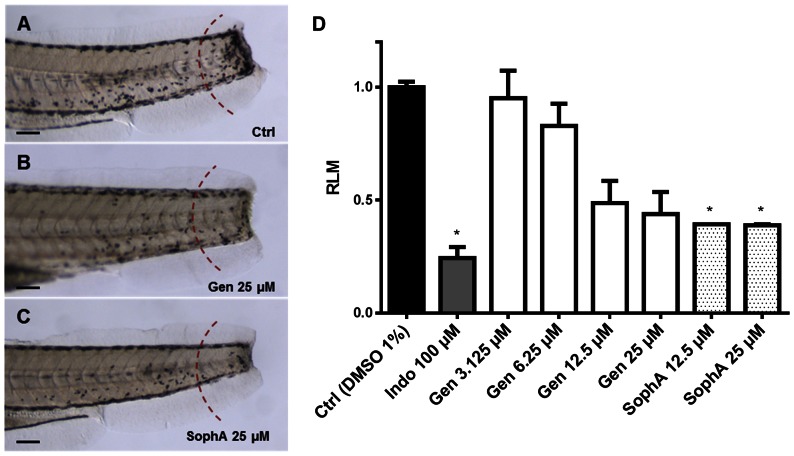
Anti-inflammatory effect of genistein and sophoraisoflavone A. **A** to **C**, zebrafish larvae are 4 dpf (days post-fertilization) with anterior to the left, scale bar = 10 µm. Migrating leukocytes were counted on one side in the tail in the region to the right of the dashed red arc and migration values were expressed as relative leukocyte migration (RLM) (C). **A**, negative control (DMSO 1%); **B**, genistein 25 µM; **C**, sophoraisoflavone A 25 µM; **D**, graph displaying the RLM in 4 dpf larvae (n = 10) after treatment with genistein and sophoraisoflavone A. RLM ≤0.5 was established as cutoff for anti-inflammatory activity. * *p*<0.05.

Since the crude extract also exhibited anti-inflammatory activity, compounds **a** to **d** were also assessed using the LPS-enhanced leukocyte migration assay in zebrafish larvae**.** Moderate but significant inhibition of leukocyte migration was observed for genistein and sophoraisoflavone A at 12.5 and 25 µM (Figure 6B–C). Intriguingly, no significant anti-inflammatory activity was observed for licoisoflavone A or rhynchoviscin, indicating some structure-dependent activity differences between these related compounds (data not shown).

Genistein, an isoflavone synthesized by Fabaceae species and usually derived from soybeans, inhibits the tyrosine kinases EGFR (epidermal growth factor receptor), pp60^v−src^, and pp110^gag−fes^ at pharmacological doses, with negligible effects against serine/threonine kinases such as protein kinase A, protein kinase C, and phosphodiesterase [36]. With regard to its role in inflammation, genistein inhibits LPS-induced nitrite production by cultured macrophages and protects against LPS-induced necrosis by reducing nitric oxide release via the downregulation of inducible nitric oxide synthase [37]. Genistein also inhibits leukocyte-endothelium interaction, thereby modulating vascular inflammation, and reduces reactive oxygen species (ROS) by attenuating the expression of ROS-producing enzymes [38].

Regarding its role in angiogenesis, genistein as well as other isoflavones are known to inhibit mammalian endothelial cell proliferation and migration *in vitro* [39], [40]. *In vivo*, genistein has been found to inhibit angiogenesis in mouse models of melanoma and breast cancer [41] and to inhibit retinal neovascularization, as well as to downregulate vascular endothelial growth factor (VEGF) and hypoxia-inducible factor (HIF1α) expression, in a mouse model of oxygen-induced retinopathy [42].

To date, no anti-angiogenic or anti-inflammatory activity has previously been reported licoisoflavone A and sophoraisoflavone A.

In the initial screen, the inhibition of angiogenesis was dependent on the original amount of each constituent in the extract. The qNMR results enable the correlation of compound amounts with bioactivity. For genistein, the analysis revealed the anti-angiogenic activity of each microfraction to correlate well with its calculated amount and thus the bioactivity profile in the initial screen had a direct quantitative link with this compound. For compounds **b**, **c** and **d**, similar activities were observed in the primary screen for microfractions containing these pure compounds, and these results were consistent with the subsequent IC_50_ analysis for each molecule – indicating the ability of this *in vivo* approach to identify microgram-level quantities of NPs possessing only moderate levels of bioactivity.

### *De Novo* Identification of the Novel Compound b

During the first phase of dereplication and microfractionation compound **b** could not be identified. Given that no phytochemical analysis has been reported for *R. viscosa* and that the anti-angiogenic activity of **b** was moderate, large scale isolation using a MS-targeted fractionation yielded 420 µg of **b**. The NMR spectra of **b** obtained from the large scale isolation matched the ones obtained during the first microfractionation. A splitting of some of the NMR signals was indicative of the possible presence of two isomers. Attempts to separate these two isomers using high-resolution isocratic conditions were not fruitful and structure identification was thus performed on the mixture by extensive 2D and ^13^C NMR spectroscopy (Figure S1).

Proton and carbon signals were assigned with the help of ^1^H, COSY, HSQC, HMBC (short and long range) and APT experiments recorded in deuterated DMSO (DMSO-*d_6_*).

The ^1^H NMR spectrum showed signals of two ^1^H pairs of a 4-oxy-phenyl group at δ_H_ 7.20/7.24 (d, *J = *8.6 Hz) and 6.74 (d, *J = *8.6 Hz), a tetra-substituted phenyl ring with the two proton signals at δ_H_ 6.09 (d, *J = *1.4 Hz) and 5.96 (d, *J = *1.4 Hz), a penta-substituted aromatic ring with a proton at δ_H_ 5.94, a dihydrofuran ring substituted by two tertiary methyl and a secondary methyl group (δ_H_ 0.94/0.97 (3H, s), 1.18/1.21 (3H, s), 1.24/1.26 (3H, d, *J* = 6.4 Hz) and 4.40/4.47 (1H, q, *J* = 6.4 Hz)) and five hydroxyl groups (δ_H_ 6.19/6.23 (1H, s), 9.38 (1H, brs), 9.63 (2H, brs) and 12.09 (1H, brs)). These signals were consistent with the skeleton of a benzodihydrofuran fused to a benzodihydropyran with a phenyl ring attached to the junction between furan and pyran ring (Figure 4). This skeleton has been found in biflavonoids from *Daphne giraldii* [43].

A long-range HMBC experiment showing a correlation between the carbon C-2'' and the hydroxyl group 3-OH as well as H-6'' and H-4'' protons confirmed that the tetra-substituted ring is linked to the dihydrofuran with the hydroxyl group 3-OH. On the other side, ^3^ *J*_CH_ HMBC correlations between carbon C-6 with H-8 and the tertiary methyl groups attached the dihydrofuran to the penta-substituted aromatic ring. Its linkage in position 6, 7 (instead of 5, 6) was confirmed by the downfield shift of the hydroxyl proton (5-OH) at δ_H_ 12.09 indicating a hydrogen bridge between 5-OH and the carbonyl C-4.

Several peaks (H-5''', H-4''', H-3''', H-2''', H-6'', 3-OH, H-2', H-6') were doubled and the carbon atoms affected were located on the methylated dihydrofuran ring (C-1''' – C-5'''), the phenol moiety (C-1' – C-6') and the bridged carbon atoms between the dihydropyran and the dihydrofuran ring (C-2 and C-3). This could indicate that stereoisomerism is located at the bridge between the dihydropyran and the dihydrofuran rings as observed for similar biflavonoids where the structure was established by X-ray on the co-crystals of the stereoisomeric mixture [43]. Thus, **b** corresponds to a very rare skeleton and this new compound was named rhynchoviscin; its structure as well as the ones of **a**, **c**, **d** and **e** are given in Figure 4.

### Conclusion

The known anti-inflammatory and anti-angiogenic activities of genistein provide an initial validation of our NP discovery approach. We used *in vivo* zebrafish-based assays to screen crude plant extracts and subsequently, perform UHPLC-PDA-TOFMS profiling and bioassay-guided microfractionation to isolate the bioactive constituents of *R. viscosa*. These were then structurally elucidated via high-resolution MS and microflow NMR.

Applying this generic miniaturized procedure, the phytochemical analysis and the generation of microfractions for biological evaluation of an NP extract and its individual constituents is feasible within one day. An initial evaluation of the biological profile of a given NP extract and its constituents is therefore achievable within approximately one week in high-content zebrafish-based bioassays.

This strategy represents a substantial acceleration of the NP-based drug discovery process and allows valuable resources required for the isolation of larger amounts of bioactive molecules for testing in mice to be dedicated only towards extracts having already demonstrated promising bioactivity *in vivo* at the microgram scale.

The key advantages of this approach are the microgram scale at which both biological and analytical experiments can be performed and the speed and the rationality of the bioassay-guided fractionation, which are generic for NP extracts of diverse origin, and require only limited sample-specific optimization [44]. Moreover, TOFMS and microflow NMR data enable dereplication early in the NP discovery process, and the systematic use of *in vivo* assays enables the identification of natural products with novel bioactivities that to date could not readily be determined through traditional assays.

In addition to genistein, bioactive constituents of *R. viscosa* included licoisoflavone A and sophoroisoflavone A – isoflavone derivatives that are structurally closely related. The novel compound identified by this study, rhynchoviscin, indicates the potential of this integrated approach to also identify bioactive NPs that occur only in limiting quantities, and which have only moderate bioactivity. Overall, these initial results demonstrate the potential of zebrafish bioassay-guided microfractionation, in combination with high-resolution MS and sub-milligram NMR techniques, to rapidly identify bioactive NPs and to quantitatively determine their *in vivo* bioactivity.

## Materials and Methods

### Ethics Statement

Permission to collect *R. viscosa* was granted by the Muhimbili University of Health and Allied Sciences in Dar es Salaam, Tanzania. Permission by local or federal government authorities was not required to collect this species on public land. Furthermore, as *R. viscosa* is not a protected or endangered species, the collection of this species for any purpose, including for scientific research, is not regulated.

All animal procedures were performed in accordance with Belgian and European Laws, guidelines and policies for animal experimentation, housing and care (Belgian Royal Decree of 6 April 2010 and European Directive 2010/63/EU on the protection of animals used for scientific purposes of 20 October 2010). This project was approved by the Animal Ethics Committee of the University of Leuven (approval number P101/2010).

### General Experimental Procedures

Molar extinction coefficients were determined on a Perkin Elmer UV/VIS Lambda 20 spectrometer and calculated based on the quantities determined by NMR.

### Chemicals & Compounds

Solvents used for sample preparation were MeOH from VWR (HiPerSolv CHROMANORM), ultrapure water (Direct-Q 3 UV water purification system, Millipore), and dichloromethane (DCM, VWR). For the HPLC isolation step, solvents were HPLC grade MeOH Chromanorm from VWR, formic acid (FA, 98%) from Fluka and ultrapure water (Millipore). ULC/MS grade MeOH, acetonitrile (ACN), H_2_O and FA (99%) from Biosolve was used for the UHPLC-PDA-TOFMS analyses. For the NMR experiments, methanol-*d_4_* (99.8% atom deuterium), acetone-*d_6_* and DMSO-*d_6_* (99.9% atom deuterium) was obtained from Armar Chemicals and Cambridge Isotope Laboratories Inc. respectively. Genistein (99% pure) was obtained from Acros Organics and maleic acid (ReagentPlus® >99.0%) from Sigma-Aldrich. For the bioassays, 1-phenyl-2-thiourea (PTU) and tricaine (ethyl 3-aminobenzoate) were purchased from Sigma-Aldrich, DMSO from Acros Organics.

### Plant Material, Extraction, Prepurification

*Rhynchosia viscosa* (Roth) DC. was collected on public land in Tabora, Tanzania and a voucher specimen (number HOS 3119) was deposited at the Faculty of Pharmacy of the Muhimbili University of Health and Allied Sciences (MUHAS), Dar es Salaam, Tanzania. The plant material was dried at room temperature and ground. The dry, powdery plant sample was exhaustively extracted with MeOH by maceration. The dry methanolic extract was obtained after removing the solvent by evaporation under reduced pressure. Prior to testing, an aliquot of the dry methanolic extract was suspended in 100% DMSO; this stock solution was then kept at -20°C.

The crude methanolic extract of *R. viscosa* was dissolved in 80% aq. MeOH and purified by SPE (ZEOprep 60, C_18_, 40-63 µm, Zeochem AG) using 80% aq. MeOH. Then, the sample was solubilized in 95% aq. MeOH and eluted over a polyamide-filled cartridge with 95% aq. MeOH that was pre-conditioned with MeOH and 95% aq. MeOH [45] to remove tannins from the extract. The sample was evaporated to dryness under reduced pressure and a reddish solid as well as an orange oil was obtained. This sample was extracted with DCM for enrichment and the remaining part was used for microfractionation.

### Microfractionation by Semi-preparative LC-MS

The enriched extract (19.8 mg) was redissolved in pure MeOH, filtered over a 0.45 µm Nylon 66 syringe filter (BGB Analytik AG) and fractionated by means of semi-preparative HPLC. The gradient method was transferred using HPLC Calculator v3.0 [28]. The separation was accomplished on a Varian modular HPLC system with a Varian 9012 pump coupled through a Thermo Scientific electrospray ionization (ESI) interface to an ion trap mass spectrometer instrument (LCQ, Thermo Scientific) and a UV detector (at 254 nm, 2151 variable wavelength monitor, LKB Bromma) to monitor the separation. A splitter enabled 50 µL/min of the flow coming from the HPLC to enter the mass spectrometer. The following negative ionization-ESI (NI-ESI) conditions were used: capillary temperature, 200°C; capillary voltage, −38 V; spray voltage, 3 kV; tube lens offset, −3 V. The acquisitions were performed in NI mode using a full scan mode over an *m/z* range of 150–1000. An in-source fragmentation energy of 5 V was applied. The separation was performed on a 250×10 mm i.d., 5 µm, XBridge™ BEH C_18_ column (Waters) in gradient mode at 2.3 mL/min with the following solvent system: A = 0.1 vol% FA-H_2_O, B = 0.1 vol% FA-MeOH; 40% B for 3.4 min and 40–90% B in 74.7 min and 90% B for 12 min. The injected volume was 500 µL. Fractions of 1.15 mL were collected every 30 s with a Gilson FC204 Fraction Collector directly into conical-bottom 96-deepwell plates (VWR). An aliquot of each microfraction (115 µL; 10% of the total microfraction volume, aliquot A) from the semi-preparative isolation step was transferred to a 96-well plate (Nunc, V96, PP, 0.45 mL), dried in a vacuum centrifuge (Genevac HT-4X, Genevac Inc.) and used for bioactivity testing in zebrafish. Another aliquot of each microfraction (11.5 µL; 1% of the total microfraction volume, aliquot B) was transferred to a 96-well plate (Nunc, V96, PP, 0.45 mL), diluted to 200 µL with 85% aq. MeOH, sealed and stored at 5°C for further purity check by UHPLC-PDA-TOFMS.

### UHPLC-PDA-TOFMS Experiments

UHPLC-PDA-TOFMS analyses were performed using an Acquity™ UPLC chromatograph and a Micromass-LCT Premier Time of Flight mass spectrometer equipped with an ESI interface (Waters). For the profiling of the crude extract, analyses on the generic gradient method were performed using a 150×2.1 mm i.d., 1.7 µm, Acquity BEH C_18_ UPLC column (Waters). For the optimized gradient method, a 100×2.1 mm i.d., 1.7 µm, Acquity BEH C_18_ UPLC column (Waters) was used and for the verification of the purity and identity of the microfractions, a short analysis was performed on a 50×2.1 mm i.d., 1.7 µm, Acquity BEH C_18_ UHPLC column (Waters). The analysis conditions are given in detail in the Text S1.

### Dereplication Procedure

The procedure published by Funari et al. [29] was used for the dereplication of compounds in the crude extract and identification of the isolated compounds. For the database search (DNP, SciFinder), hits were refined by searching for compounds isolated from Fabaceae species. More details on the dereplication procedure are given in the Text S1.

### Quantitative Microflow NMR Measurements

NMR spectra of the microfractions were recorded on a Varian INOVA 500 MHz NMR instrument at 25°C, equipped with a microflow NMR probe (CapNMR™) and an automated sample injection unit (One Minute-NMR™) from Protasis. Remaining amounts of microfractions (89% of the total microfraction volume, aliquot C) were diluted in 10 µL of methanol-*d_4_* whereof 8 µL were injected.

For the quantitative studies, the relaxation delay *T*_1_ was experimentally determined for all protons of genistein to choose the recycle delay for qNMR acquisition and to determine the ^1^H signals suitable for quantification. The protons on cycle B (*T*_1_ = 2.0–2.3 s) and C (*T*_1_ = 2.7 s) were fully recovered (time >5*T_1_) within a recycle delay of less than 15 s, whereas the protons on cycle A were only fully recovered after 25 s. A recycle delay of 20 s was set for qNMR experiments and well resolved ^1^H signals on cycle B were chosen for quantification. The optimal pulse width at 90° was arrayed (at 360°) for every individual sample and lays between 4.1 and 4.2 µs. FIDs were Fourier transformed with LB = 0.3 Hz. The resulting spectra were manually phased, baseline corrected using a 1^st^ order polynomial function and calibrated to the residual methanol peak at 3.31 ppm using MestReNova (version 6.01, Mestrelab Research S.L.) The signals were integrated manually and the concentration was determined using PULCON [17]. Maleic acid was used as external standard.

### Zebrafish

The transgenic line *fli-1*:EGFP [23] was obtained from the Zebrafish International Resource Center at the University of Oregon (Eugene, Oregon, USA). Zebrafish husbandry, embryo collection, and embryo and larva maintenance were performed as previously described [46], [47]. For the leukocyte migration assay, zebrafish embryos at one day post fertilization (dpf) were exposed to 1-phenyl-2-thiourea (PTU) to suppress melanization (Text S1). For this assay and for confocal imaging, larvae were anesthetized with tricaine (Text S1).

The leukocyte migration assay was performed in 24-well microtiter plates using ten 4 dpf larvae per well in 1 mL of Danieau’s medium (Text S1). The vascular outgrowth assay was performed in 96-well microtiter plates using five embryos at 16 hours post-fertilization (hpf) per well in 200 µL of Danieau’s medium. Extracts and compounds were solubilized in DMSO, and were added to the Danieau’s medium up to a maximum DMSO concentration of 1%.

### Anti-inflammatory Assay

Prior to assessment of the anti-inflammatory activity of *R. viscosa* and its derivatives, *in vivo* toxicological tests were performed to establish the maximum tolerated concentration of each sample (Text S1). Next, a LPS-enhanced leukocyte migration assay was performed. Briefly, larvae were pre-incubated (1 hour at 28°C, ±0.5) with specific concentrations of each sample. Negative controls, containing only vehicle (1% DMSO), and positive controls, indomethacin 50–100 µM, were processed in parallel. After pre-incubation, larvae were anesthetized and subjected to complete tail transection made 0.5 mm (±0.2) from the tip of the tail of each larvae under microscopy light (Carl Zeiss Stemi 2000C) using a scalpel. Next, tail-cut larvae were briefly rinsed in Danieau’s medium without tricaine and incubated for seven hours with specific concentrations of each sample containing 10 µg/mL LPS (*Salmonella typhosa* ATCC 10749, Sigma-Aldrich). After this incubation, larvae were fixed in 4% paraformaldehyde and kept overnight at 4°C. Fixed larvae were gently washed with PBST (PBS-1X phosphate buffered saline, Gibco +0.1% Tween 20) and next subjected to incubation (15 minutes at room temperature) with 1 mL of freshly prepared staining solution (Leucognost® Pox, Merck). Evaluation of the migrating leukocytes to the injured region was done in one side of each larva under light microscopy and scoring of the migration was assessed according to a 5-point index of staining intensity. The average of these values for each experimental group were normalized against the average values of the control group (1% DMSO) and expressed as RLM, which for significant anti-inflammatory activity has a cutoff point of RLM ≤0.5. All experiments were performed in duplicate, with ten larvae per condition. Statistical analysis was done using GraphPad Prism 5 software using one-way analysis of variance (ANOVA).

### Angiogenesis Assay

Prior the initiation of ISV outgrowth, *fli*-1:EGFP embryos at 16 hpf were incubated (32 hours at 28°C, ±0.5) with specific concentrations of extracts and compounds. Negative controls, containing only vehicle (1% DMSO) were processed in parallel. The microfraction samples for biological profiling (aliquot A of each microfraction) were dried, re-solubilized in 3 µL DMSO and diluted to 150 µL with Danieau’s medium, of which 90 µL were used for a first screen. Microfractions with 100% inhibitory activity or exhibiting toxicity were tested at a lower concentration (1/3 of the initial concentration).

Inhibition of vascular outgrowth along the trunk of every larva was evaluated under UV microscopy light (MZ10F Leica stereo microscope) at 48 hpf and scoring of anti-angiogenic activity was done according to a 5-point index for vascular outgrowth. The average of the values for each experimental group was normalized against the average of the values of the control group (1% DMSO), yielding a relative vascular outgrowth (RVO) score that was then expressed as percentage of inhibitory activity. All experiments were performed in duplicate, with five larvae per condition. Statistical analysis and IC_50_ curves were done using GraphPad Prism 6 software using nonlinear regression to fit the data to the log (inhibitor) vs. response curve (variable slope). Representative embryos were subjected to confocal imaging (see below).

### Confocal Imaging

Confocal imaging (Figure 2 and 5) was carried out using a Nikon A1R confocal unit (Nikon) mounted on a Ti2000 inverted microscope (Nikon). For the imaging, 4×(0.2 N.A.) and 10×(0.45 N.A.) lenses were used. For detecting the fluorescence of the fish embryos, a 488 nm laser line (CVI Melles Griot) and detection filters for the range of 515–550 nm were used. Confocal stacks of the whole fish or the depicted regions were acquired and projections of the maximum intensity of the 3D volume shown. During imaging, zebrafish embryos were anesthetized using 0.1 mg/mL tricaine in Danieau’s medium.

### Novel Compound from *Rhynchosia viscosa* with Anti-angiogenic Activity

Rhynchoviscin (**b**). Insufficient material was available to obtain an optical rotation value. Purity: 80% (determined by NMR). UV (MeOH) λ_max_ (log ε) 304 nm (4.39);^ 1^H NMR (DMSO-*d_6_*, 500 MHz, CapNMR™ probe, δ_H_): 0.94/0.97 (3H, s, H-5'''), 1.18/1.21 (3H, s, H-4'''), 1.24/1.26 (3H, d, *J* = 6.4 Hz, H-3'''), 4.40/4.47 (1H, q, *J* = 6.4 Hz, H-2'''), 5.90 (1H, d, *J* = 1.4 Hz, H-4''), 5.99 (1H, s, H-8), 6.03/6.04 (1H, d, *J* = 1.4 Hz, H-6''), 6.19/6.23 (1H, s, 3-OH), 6.74 (2H, d, *J* = 8.6 Hz, H-3'/H-5'), 7.20/7.24 (2H, d, *J* = 8.6, H-2'/H-6'), 9.38 (1H, brs, 4'-OH), 9.63 (2H, brs, 3''-OH/5''-OH), 12.09 (1H, brs, 5-OH). ^13^C NMR (DMSO-*d6*, 500 MHz, CapNMR™ probe, δ_C_): 13.8/14.3 (C-3'''), 20.8 (C-5'''), 24.5/25.4 (C-4'''), 42.6 (C-1'''), 80.3 (C-3), 90.4 (C-6''), 90.4/90.6 (C-2'''), 91.4/91.5 (C-8), 97.3 (C-4''), 99.8 (C-4a), 105.6 (C-2''), 113.0 (C-6), 114.5 (C-3'/C-5'), 117.1 (C-2), 124.6 (C-1'), 127.9/128.1 (C-2'/C-6'), 155.5 (C-3''), 158.2 (C-4'), 161.0 (C-5''), 161.5 (C-1''), 163.9 (C-8a), 167.0 (C-7), 192.8 (C-4). ESI-MS (NI mode): *m/z* 477.1195 [M-H]^–^ (C_26_H_22_O_9_, calc. *m/z* 477.1186, Δ 1.9 ppm).

Detailed structure information on compound **a**, **c**, **d** and **e** can be found in the Text S1. NMR spectra for rhynchoviscin are given in Figure S1.

## Supporting Information

Figure S1 **NMR Spectra (1H, APT, HSQC, HMBC) of Rhynchoviscin.**
(PDF)

Text S1 **Supplementary Information on Materials & Methods.**
(DOC)

Text S2 **Supplementary Information on Quantitative Microflow NMR.**
(DOC)
