# Supplementary material for: Integration of Microfractionation, qNMR and Zebrafish Screening for the In Vivo Bioassay-Guided Isolation and Quantitative Bioactivity Analysis of Natural Products
Source: PLoS One. 2013 May 21;8(5):e64006. doi: 10.1371/journal.pone.0064006 (PMC3660303; doi:10.1371/journal.pone.0064006)
Supplement: Figure S1 — NMR Spectra (1H, APT, HSQC, HMBC) of Rhynchoviscin. (PDF) [file pone.0064006.s001.pdf]

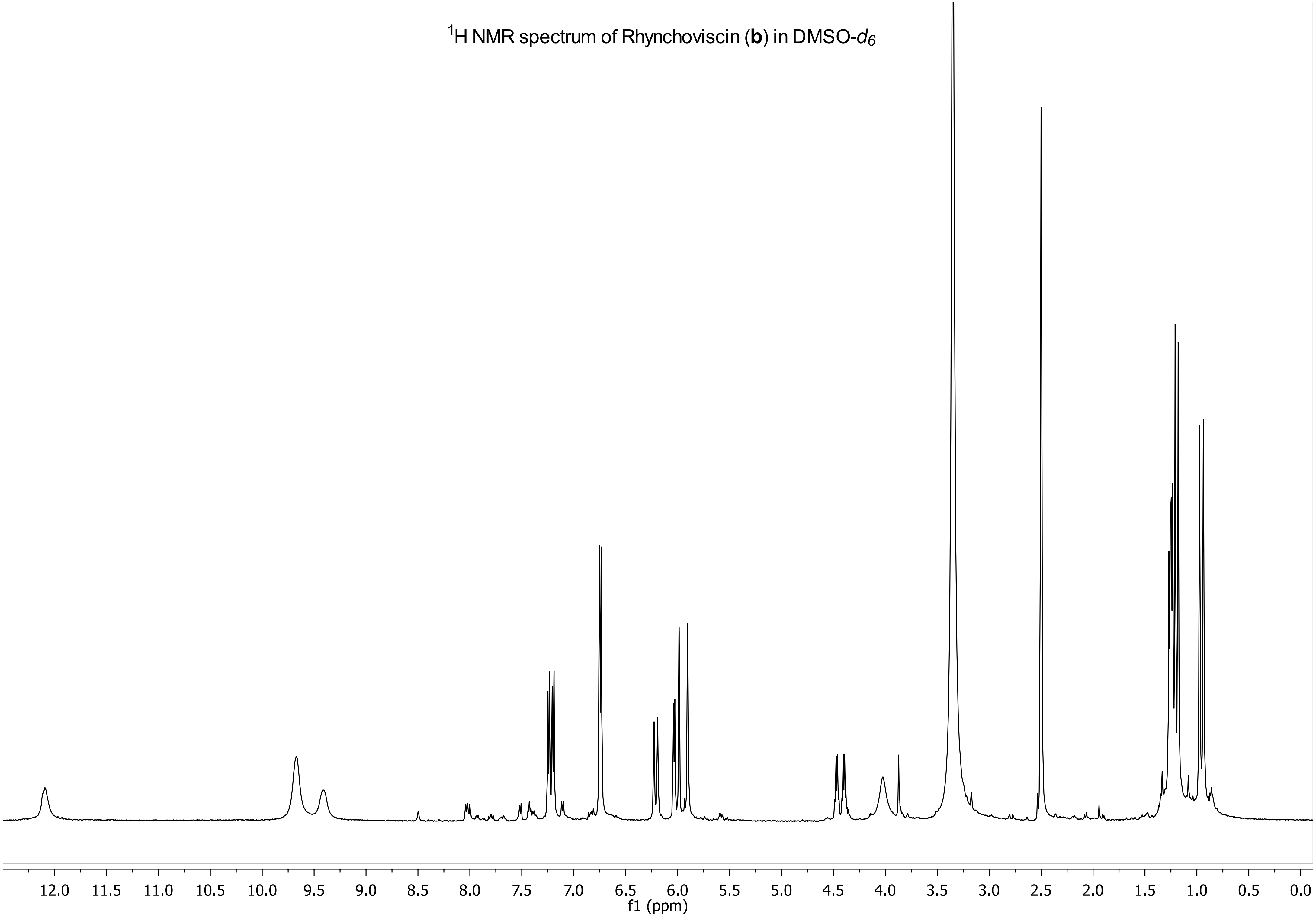

^{1}H NMR spectrum of Rhynchoviscin (**b**) in DMSO-d_6
12.0 11.5 11.0 10.5 10.0 9.5 9.0 8.5 8.0 7.5 7.0 6.5 6.0 5.5 5.0 4.5 4.0 3.5 3.0 2.5 2.0 1.5 1.0 0.5 0.0
f1 (ppm)

^{13}C NMR spectrum (APT) of Rhynchoviscin (**b**) in DMSO-d_6

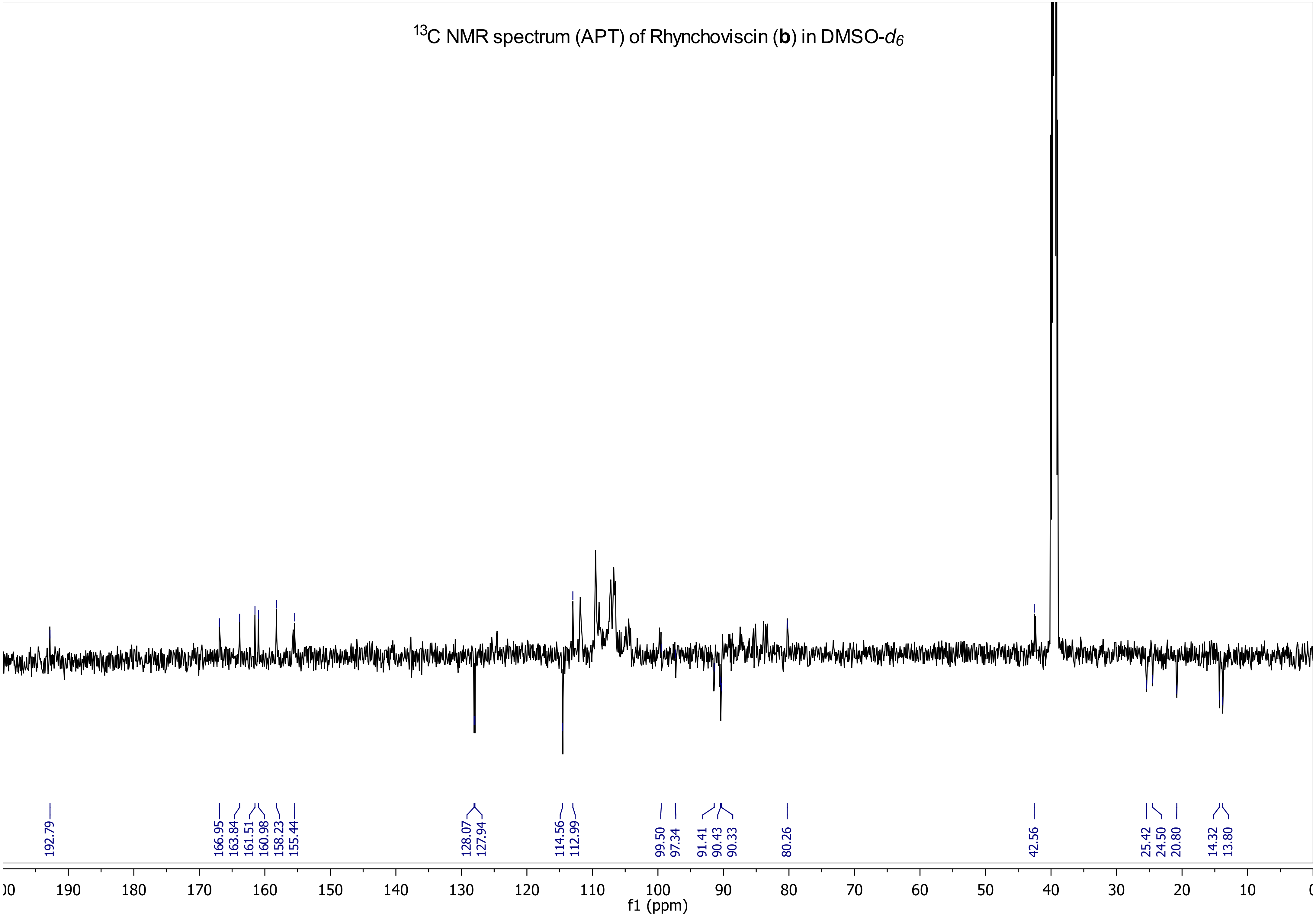

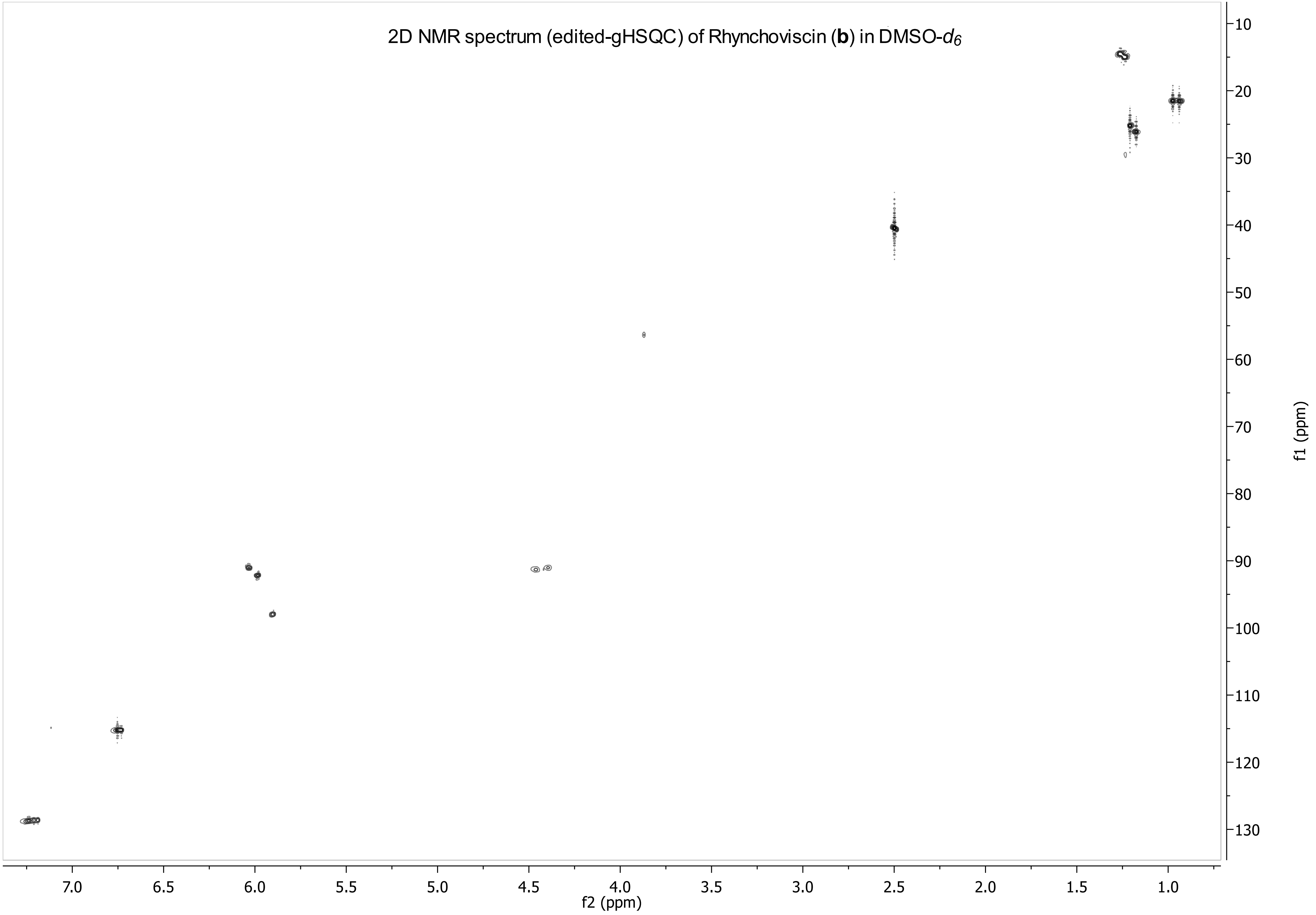
2D NMR spectrum (edited-gHSQC) of Rhynchoviscin (**b**) in DMSO-d_6
f2 (ppm)
f1 (ppm)
7.0
6.5
6.0
5.5
5.0
4.5
4.0
3.5
3.0
2.5
2.0
1.5
1.0
10
20
30
40
50
60
70
80
90
100
110
120
130

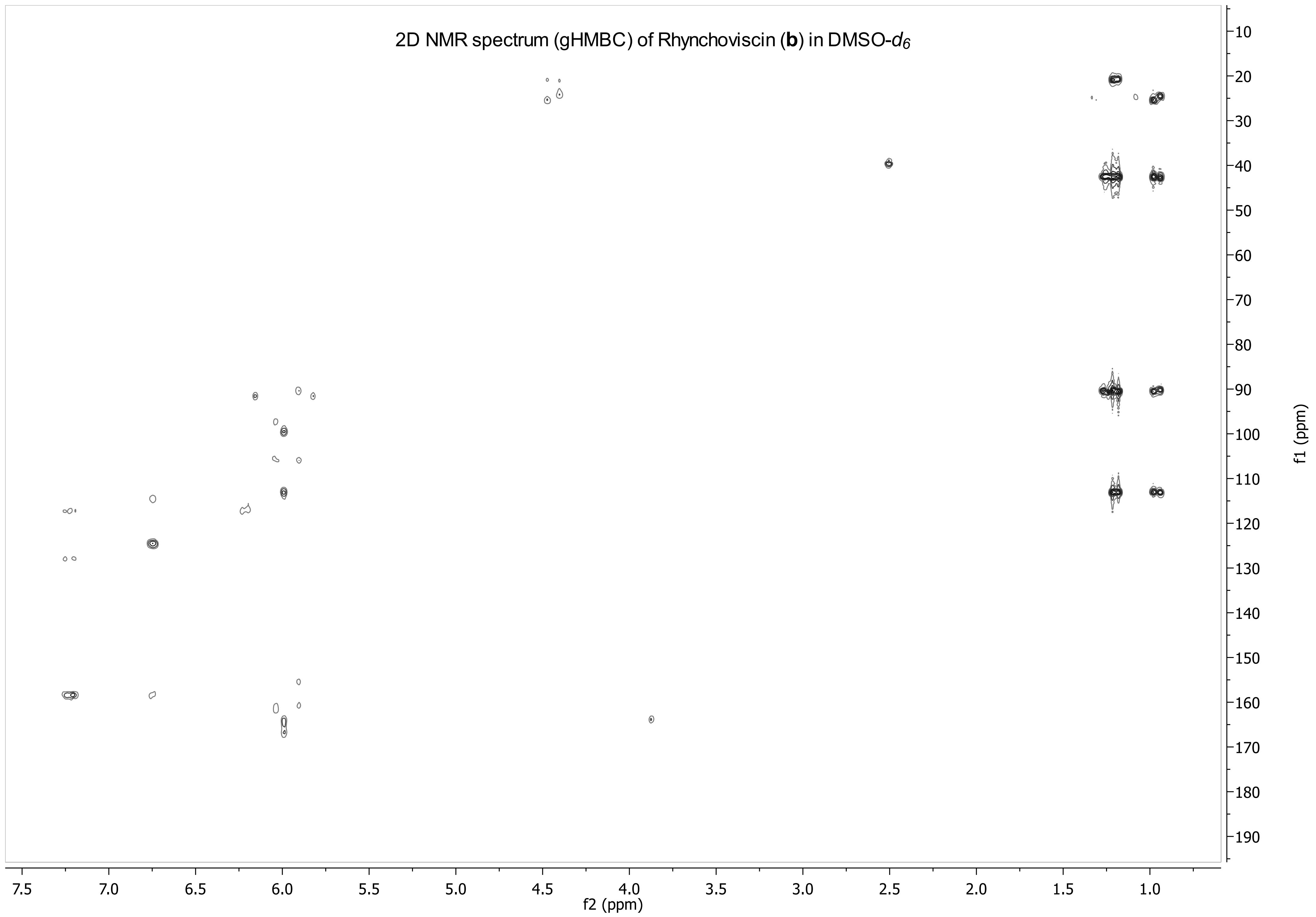
2D NMR spectrum (gHMBC) of Rhynchoviscin (b) in DMSO-d6
f2 (ppm)
f1 (ppm)
7.5 7.0 6.5 6.0 5.5 5.0 4.5 4.0 3.5 3.0 2.5 2.0 1.5 1.0
10 20 30 40 50 60 70 80 90 100 110 120 130 140 150 160 170 180 190
