# Supplementary material for: Integration of Microfractionation, qNMR and Zebrafish Screening for the In Vivo Bioassay-Guided Isolation and Quantitative Bioactivity Analysis of Natural Products
Source: PLoS One. 2013 May 21;8(5):e64006. doi: 10.1371/journal.pone.0064006 (PMC3660303; doi:10.1371/journal.pone.0064006)
Supplement: Text S1 — Supplementary Information on Materials & Methods. (DOC) [file pone.0064006.s002.doc]

**Text S1 - Supplementary Information on Materials and Methods**

**Supporting information for:**

**Integration of Microfractionation, qNMR and Zebrafish Screening for the *In Vivo* Bioassay-Guided Isolation and Quantitative Bioactivity Analysis of Natural Products**

Nadine Bohni, María Lorena Cordero-Maldonado, Jan Maes, Dany Siverio-Mota, Laurence Marcourt, Sebastian Munck, Appolinary R. Kamuhabwa, Mainen J. Moshi, Camila V. Esguerra, Peter A. M. de Witte, Alexander D. Crawford, Jean-Luc Wolfender

## UHPLC-PDA-TOFMS Experiments

UHPLC‑PDA‑TOFMS analyses for the profiling of the crude extract were performed using an Acquity™ UPLC chromatograph and a Micromass-LCT Premier Time of Flight mass spectrometer equipped with an ESI interface (Waters). For the generic gradient method, detection was performed in positive ionization (PI) and NI mode in the range *m/z* 100-1000 in centroid mode with a scan time of 0.3 s. ESI conditions were: capillary voltage 2800 V, cone voltage 40 V, MCP detector voltage 2450 V, source temperature 120 °C, desolvation temperature 300 °C, cone gas flow 20 L/h, and desolvation gas flow 800 L/h. The mass spectrometer was internally calibrated by infusion of a solution of leucine-enkephalin (2 μg/mL, Sigma-Aldrich) through the lockmass spray probe at a flow rate of 10 μL/min, using a second Shimadzu LC-10ADvp LC pump. The separation was performed on a 150 x 2.1 mm i.d., 1.7 μm, Acquity BEH C_18_ UPLC column (Waters) in the gradient mode at a flow rate of 0.46 mL/min with the following solvent system: A = 0.1 vol% FA-H_2_O, B = 0.1 vol% FA-ACN; 5–95% B in 30 min. The injected volume was 2 μL. The PDA traces were recorded from 210 to 450 nm.

For the optimized gradient method, detection was performed in negative ion (NI) mode as above. ESI conditions in NI mode were: capillary voltage 2800 V, cone voltage 40 V, MCP detector voltage 2400 V, source temperature 120 °C, desolvation temperature 300 °C, cone gas flow 20 L/h, and desolvation gas flow 700 L/h. The separation was performed on a 100 x 2.1 mm i.d., 1.7 μm, Acquity BEH C_18_ UPLC column (Waters) in the gradient mode at a flow rate of 0.306 mL/min with the following solvent system: A = 0.1 vol % FA-H_2_O, B = 0.1 vol % FA-MeOH; 40–90% B in 11.4 min. The injected volume was 1 μL.

For the verification of the identity and purity of the microfractions, a short analysis was performed on a 50 x 2.1 mm i.d., 1.7 μm, Acquity BEH C_18_ UHPLC column (Waters) in the gradient mode at a flow rate of 0.3 mL/min with the following solvent system: A = 0.1 vol% FA-H_2_O, B = 0.1 vol% FA-ACN. The injected volume was 1 μL, 1% of the microfraction was taken and diluted to 200 μL with H_2_O:ACN 1:1 + 0.1 vol% FA.

## Dereplication Procedure

The procedure published by Funari et al. was used for the dereplication of compounds in the crude extract and identification of the isolated compounds. Briefly, possible molecular formulae were calculated in MassLynx™ (Waters) using a mass tolerance of 15 ppm, allowing atoms C, H, O and N with no restrictions. These formulae were refined by applying heuristic filtering (seven golden rules ) and only molecular formula reported from natural origin and isolated from plant sources were considered. Furthermore, database hits were refined by searching for compounds isolated from Fabaceae species. For molecules exhibiting the typical isoflavone PDA absorption, the isoflavone skeleton was used to restrict the database search (DNP, SciFinder).

## Zebrafish Procedures

### Danieau’s Medium

Danieau’s medium was used for maintenance of newly collected zebrafish embryos and for all experimental incubations. A stock solution (30X consisting of 1.74 M NaCl, 21 mM KCl, 150 mM Hepes buffer (pH 7.1-7.3), 18 mM Ca(NO_3_)_2_ and 12 mM MgSO_4_) was prepared in advance and kept at room temperature. The working solution – 0.3X Danieau’s medium – prepared as a 1/100 dilution of the stock solution in ultrapure water, included methylene blue (0.03M) to avoid fungus and bacterial growth. This working medium was kept at room temperature.

### Treatment with 1-Phenyl-2-thiourea (PTU)

For inhibition of melanocytes that can interfere with visual assessment of the stained migrating leukocytes, zebrafish embryos were treated with PTU. A stock solution (2 mM PTU) was prepared in ultrapure water in advance and kept at room temperature until needed. At one day post-fertilization, 1/10 dilution of the stock solution in Danieau’s is used as a medium for embryos maintenance. To ensure proper inhibition of melanocytes, PTU was exchanged daily until the tail cut assay was performed at four days post-fertilization.

### Treatment with Ethyl 3-aminobenzoate (Tricaine)

To reduce animal discomfort during experimental procedures, zebrafish larvae were anesthetized using tricaine. A stock solution (10 mg/mL tricaine) was prepared in ultrapure water in advance and kept at 4°C until needed. Larvae subjected to tail cut and to confocal imaging were immersed in 1/100 dilution of the stock solution in Danieau’s medium.

### Toxicological Evaluation

Prior to assessment of the anti-inflammatory activity, *in vivo* toxicological tests were performed to establish the maximum tolerated concentration of each sample. Zebrafish larvae at 4 dpf were treated with different concentrations of the extract and isolated compounds, and incubated at 28 °C (± 0.5). For the next eight hours, hourly microscopic examination of the larvae was done to determine signs of toxicity, e.g. cardiovascular defects (arrhythmia or decreased circulation), balance defects (loss of posture), locomotor defects (decreased touch response) or death. Concentrations inducing any of these effects were not considered for testing anti-inflammatory activity.

## Isolated Compounds from *Rhynchosia viscosa* with Anti-angiogenic Activity

Genistein (**a**). ^1^H NMR (methanol-*d_4_*, 500 MHz, CapNMR™ probe, δ_H_): 6.23 (1H, d, *J* = 2.1 Hz, H-6), 6.34 (1H, d, *J* = 2.1 Hz, H-8), 6.85 (2H, d, *J* = 8.8 Hz, H-3'/H-4'), 7.37 (2H, d, *J* = 8.8 Hz, H-2'/H‑5'), 8.05 (1H, s, H-2). ESI-MS (NI mode): *m/z* 269.0461 [M-H]^–^ (C_15_H_10_O_5_, calc. *m/z* 269.0450, Δ 4.1 ppm). These data were identical to literature values and those obtained on a commercial sample.

Sophoraisoflavone A (**c**). UV (MeOH) λ_max_ (log ε) 259 nm (4.78); ^1^H NMR (methanol-*d_4_*, 500 MHz, CapNMR™ probe, δ_H_): 1.38 (6H, s, H-5''/H-6''), 5.60 (1H, d, *J* = 9.9 Hz, H-3''), 6.23 (1H, d, *J* = 2.1 Hz, H-6), 6.36 (1H, d, *J* = 2.1 Hz, H-8), 6.40 (1H, d, *J* = 8.3 Hz, H-5'), 6.68 (1H, d, *J* = 9.9 Hz, H-2''), 6.95 (1H, d, *J* = 8.3 Hz, H-6'), 7.93 (1H, s, H-2). ESI-MS (NI mode): *m/z* 351.0876 [M-H]^–^ (C_20_H_16_O_6_, calc. *m/z* 351.0869, Δ 2.0 ppm).

Licoisoflavone A (**d**). ^1^H NMR (methanol-*d_4_*, 500 MHz, δ_H_): 1.66 (3H, s, H-4''), 1.77 (3H, s, H-5''), 3.38 (2H, s, H-1''), 5.25 (1H, m, H-2''), 6.27 (1H, d, *J* = 2.1 Hz, H-6), 6.39 (1H, d, *J* = 2.1 Hz, H-8), 6.43 (1H, d, *J* = 8.3 Hz, H-5'), 6.85 (1H, d, *J* = 8.3 Hz, H-6'), 8.05 (1H, s, H-2). ^13^C NMR (methanol-*d_4_*, 125 MHz): δ 16.8 (C-5''), 22.5 (C-1''), 24.8 (C-4''), 93.7 (C-8), 99.3 (C-6), 107.6 (C-5'), 123.3 (C-2''), 128.1 (C-6'), 130.3 (C-3''), 154.5 (C-4'), 155.8 (C-2), 182.2 (C-4). ESI-MS (NI mode): *m/z* 353.1037 [M-H]^–^ (C_20_H_18_O_6_, calc. *m/z* 353.1025, Δ 3.4 ppm).

3’-O-Methylorobol (**e**). ^1^H NMR (methanol-*d_4_*, 500 MHz, δ_H_): 3.90 (3H, s, 3’-O-CH_3_), 6.23 (1H, d, *J* = 1.9 Hz, H-6), 6.36 (1H, d, *J* = 1.9 Hz, H-8), 6.86 (1H, d, *J* = 8.2 Hz, H-5’), 6.97 (1H, dd, *J* = 8.2, 1.2 Hz, H-6’), 7.16 (1H, d, *J* = 1.2 Hz, H-2’), 8.10 (1H, s, H-2). ESI-MS (NI mode): *m/z* 299.0549 [M-H]^–^ (C_16_H_12_O_7_, calc. *m/z* 299.0556, Δ 2.3 ppm).

**References**

1. Funari CS, Eugster PJ, Martel S, Carrupt P-A, Wolfender J-L, et al. (2012) High resolution ultra

high pressure liquid chromatography–time-of-flight mass spectrometry dereplication strategy for the

metabolite profiling of Brazilian *Lippia* species. J Chromatogr, A 1259: 167-178.

2. Kind T, Fiehn O (2007) Seven golden rules for heuristic filtering of molecular formulas obtained by

accurate mass spectrometry. BMC Bioinf 8: 105.

3. Caligiani A, Palla G, Maietti A, Cirlini M, Brandolini V (2010) ^1^H NMR fingerprinting of soybean

extracts, with emphasis on identification and quantification of isoflavones. Nutrients 2: 280-289.
