# Supplementary material for: Integration of Microfractionation, qNMR and Zebrafish Screening for the In Vivo Bioassay-Guided Isolation and Quantitative Bioactivity Analysis of Natural Products
Source: PLoS One. 2013 May 21;8(5):e64006. doi: 10.1371/journal.pone.0064006 (PMC3660303; doi:10.1371/journal.pone.0064006)
Supplement: Text S2 — Supplementary Information on Quantitative Microflow NMR. (DOC) [file pone.0064006.s003.doc]

**Text S2 - Supplementary Information on Quantitative Microflow NMR**

**Supporting information for:**

**Integration of Microfractionation, qNMR and Zebrafish Screening for the *In Vivo* Bioassay-Guided Isolation and Quantitative Bioactivity Analysis of Natural Products**

Nadine Bohni, María Lorena Cordero-Maldonado, Jan Maes, Dany Siverio-Mota, Laurence Marcourt, Sebastian Munck, Appolinary R. Kamuhabwa, Mainen J. Moshi, Camila V. Esguerra, Peter A. M. de Witte, Alexander D. Crawford, Jean-Luc Wolfender

## Quantitative Microflow NMR

For NMR quantification, a strategy which does not alter the sample by addition of an internal standard was favored so that any interference with bioassays is avoided. In this respect, a quantitative NMR (qNMR) method using an external calibration (PULCON ) was used. PULCON correlates the absolute intensities of ^1^H signals in two spectra measured in different solution conditions. Therefore, the exact pulse length (360° radio frequency pulse) was determined for every sample to account for inter-sample differences. The microflow NMR setup that includes a sample injection module is particularly suited for qNMR by PULCON. Indeed, microflow probes are known to provide high stability of the field homogeneity as the geometry of the sample is fixed and the sample fills the radio frequency coil the same way each time ensuring a stable pulse width from one sample to the other. Furthermore, the automated injection makes the loading of the sample more reproducible which increases the accuracy of the quantification. The longitudinal relaxation time (*T*_1_) for the protons of the various polyphenols was estimated by an inversion recovery experiment on different types of aromatic protons on a model isoflavone to ensure reliable quantification results (see Materials & Methods).

To verify that the method was suitable for estimation of amounts collected by microfractionation, an extract of *Lupinus albus*, known to contain genistein, was selected and microfractionated. Genistein was collected in a single microfraction and quantification results obtained by qNMR on this fraction were compared to standard HPLC-UV quantification of the corresponding LC peak in the extract. The results obtained by qNMR were 30% lower than the amount injected on column which can be explained by the recovery yield of the microfractionation procedure. The estimation of the amount by PULCON compared to a conventional method using an internal standard were similar, the latter being slightly more precise.

**References**

1. Wider G, Dreier L (2006) Measuring protein concentrations by NMR spectroscopy. J Am Chem

Soc 128: 2571-2576.

2. Schroeder FC, Gronquist M (2006) Extending the scope of NMR spectroscopy with microcoil

probes. Angew Chem Int Ed 45: 7122-7131.
